# Adherence to Gluten-Free Diet Restores Alpha Diversity in Celiac People but the Microbiome Composition Is Different to Healthy People

**DOI:** 10.3390/nu14122452

**Published:** 2022-06-14

**Authors:** Orazio Palmieri, Stefano Castellana, Antonio Bevilacqua, Anna Latiano, Tiziana Latiano, Anna Panza, Rosanna Fontana, Antonio Massimo Ippolito, Giuseppe Biscaglia, Annamaria Gentile, Domenica Gioffreda, Ivana Decina, Michele Tricarico, Milena Sinigaglia, Maria Rosaria Corbo, Tommaso Mazza, Francesco Perri, Carmela Lamacchia

**Affiliations:** 1 Division of Gastroenterology, Fondazione IRCCS “Casa Sollievo della Sofferenza”, 71013 San Giovanni Rotondo, Italy; a.latiano@operapadrepio.it (A.L.); tiziana.latiano@gmail.com (T.L.); a.panza@operapadrepio.it (A.P.); r.fontana@operapadrepio.it (R.F.); antonio.ippolito@alice.it (A.M.I.); giuseppe.biscaglia@gmail.com (G.B.); a.gentile@operapadrepio.it (A.G.); mimma_gioffreda@yahoo.it (D.G.); f.perri@operapadrepio.it (F.P.); 2 Bioinformatics Unit, Fondazione IRCCS “Casa Sollievo della Sofferenza”, 71013 San Giovanni Rotondo, Italy; s.castellana@operapadrepio.it (S.C.); t.mazza@operapadrepio.it (T.M.); 3 Department of Agriculture, Food, Natural Resources and Engineering (DAFNE), University of Foggia, 71122 Foggia, Italy; antonio.bevilacqua@unifg.it (A.B.); milena.sinigaglia@unifg.it (M.S.); mariarosaria.corbo@unifg.it (M.R.C.); carmela.lamacchia@unifg.it (C.L.); 4 New Gluten World s.r.l., 71121 Foggia, Italy; i.deci10na@gmail.com (I.D.); micheletricarico.17@alice.it (M.T.)

**Keywords:** celiac disease (CD), gluten-free diet (GFD), microbiota

## Abstract

Celiac disease (CD) is an autoimmune disease with the destruction of small intestinal villi, which occurs in genetically predisposed individuals. At the present moment, a gluten-free diet (GFD) is the only way to restore the functionality of gut mucosa. However, there is an open debate on the effects of long-term supplementation through a GFD, because some authors report an unbalance in microbial taxa composition. Methods: For microbiome analysis, fecal specimens were collected from 46 CD individuals in GFD for at least 2 years and 30 specimens from the healthy controls (HC). Data were analyzed using an ensemble of software packages: QIIME2, Coda-lasso, Clr-lasso, Selbal, PICRUSt2, ALDEx2, dissimilarity-overlap analysis, and dysbiosis detection tests. Results: The adherence to GFD restored the alpha biodiversity of the gut microbiota in celiac people but microbial composition at beta diversity resulted as different to HC. The microbial composition of the CD subjects was decreased in a number of taxa, namely Bifidobacterium longum and several belonging to Lachnospiraceae family, whereas Bacteroides genus was found to be more abundant. Predicted metabolic pathways among the CD bacterial communities revealed an important role in tetrapyrrole biosynthesis. Conclusions: CD patients in GFD had a non-dysbiotic microbial composition for the crude alpha diversity metrics. We found significant differences in beta diversity, in certain taxon, and pathways between subjects with inactive CD in GFD and controls. Collectively, our data may suggest the development of new GFD products by modulating the gut microbiota through diet, supplements of vitamins, and the addition of specific prebiotics.

## 1. Introduction

Celiac disease (CD) is an autoimmune disease with the destruction of small intestinal villi in response to gluten exposure, which occurs in genetically predisposed individuals that have specific variants of the human leukocyte antigen (HLA) DQ2 and DQ8 genes [1]. In CD patients, the ingestion of gluten, particularly wheat gliadin, leads to an exaggerated adaptive and innate intestinal immune response [1]. Currently, the common efficient treatment for CD is lifelong adherence to a strict gluten-free diet (GFD), which is able to fully restore mucosal recovery [1].

Although genetic predisposition and exposure to gluten are considered necessary triggers for CD development, further environmental factors in combination with alterations in the gut microbial composition may also contribute towards the CD pathogenesis by inducing unbalance of the intestinal homeostasis. Notwithstanding, the presence of the HLA haplotypes is necessary; they are not sufficient to promote the disease since many HLA carriers do not develop the disease. The HLA locus represents the main inherited genetic susceptibility factor for CD and accounts for ~40% of the genetic variance of the disease, while the non-MHC susceptibility loci explain ~15% of the disease risk [2].

A number of epidemiological, clinical, and animal studies suggest that broad antibiotic exposure, weaning practices [3], and viral gastrointestinal infections [4] may strongly influence the gut ecosystem. Several studies [5,6,7] show the rapid expansion of microbiota, addressing the relationship between the intestinal microbiome and CD.

Although these enormous efforts have suggested an association between altered microbiota and CD, in both pediatric and adult patients, a specific microbial signature has not been recognized probably for the presence of highly heterogeneous and sometimes contradictory studies, which return inconclusive results. The conflicting evidence among published studies could depend on patients’ age, disease phenotype, GFD, and microbiome research methods. A further important open issue remains on the role of the GFD. It is still unclear if the altered microbial composition observed in CD is related to the pathogenesis of celiac disease or is a secondary effect of nutrient deficiency or malabsorption.

Gluten exclusion is at present the only therapy to resolve CD and restore the functionality of gut mucosa. While it is known that celiac people can have a completely different quali-quantitative composition of microbiota compared to healthy people [8], there is an open debate on the effects of long-term supplementation through GFD, because some authors report an unbalance in microbiota composition also after adherence to GFD with a higher relative abundance of Proteobacteria and a lower abundance of Bacteroidetes and Firmicutes [9]. Moreover, the narrative review of Caio et al. [7] points out some taxa with an altered composition in celiac people on a GFD diet also for a long time.

Therefore, this paper aims at addressing the following questions: does GFD restore microbiota composition after a long-time adherence? Is celiac microbiota composition similar to healthy people when there are no symptoms and inflammation? Can alpha and beta-diversities describe gut microbiota in celiac people?

Therefore, with the aim to better highlight the microbiota role in CD, we analyzed the microbiota 16S rRNA by next generation-sequencing (NGS) in stool samples obtained from a well-characterized cohort of CD patients in GFD for a long time and with negative serology for CD.

## 2. Methods

*Patients*. All procedures were performed in accordance with the ethical standards of the institutional Ethics committee Casa Sollievo della Sofferenza, (decision number: 46/CE February 2017) and with the 1964 Helsinki declaration and its later amendments or comparable ethical standards. Informed consent was signed by all participants. If the patient was <18 years old, written consent to participate in the study was obtained from the parents or legal guardians.

The patients are part of a study registered on clinicaltrials.gov (Identifier: NCT03137862, accessed on 23 May 2022) and previously published by our group [10].

The control group consisted of healthy non-celiac non-hospitalized people (HC—healthy controls) including healthy subjects collected from laboratory staff and donors of stool for fecal microbiota transplantation. All donors had no previous or current history of gastrointestinal diseases or malignancies and at the sample collection and, at least 3 months earlier, were not users of probiotics or antibiotics.

*Laboratory procedures.* Fecal samples from both CD subjects and HC were collected at home and immediately frozen in the home freezer, inside a sterile container at −20 °C, and subsequently stored at −80 °C at the laboratory of CSS Hospital. Total bacterial genomic DNA was extracted by using the QIAamp PowerFecal Kit (*Qiagen*, Hilden, Germany) following the manufacturer’s recommendations. The DNA quantification, sequencing of V3 to V4 hypervariable region of the 16S rRNA gene, the quantification of gluten in stools, the symptom assessments, and the serological and genetic data of celiac patients were previously described [10].

*Bioinformatics and statistical data analyses*. The computational pipeline was composed of popular open-source tools and R packages [11] for 16S rRNA metagenomics studies.

First, QIIME2 v.2021.11 suite [12] was implemented for the first analytical steps, which included base quality control; removal of chimeric reads; paired-end reads joining; and sequence clustering, which was performed using the QIIME2 integrated DADA2 [13] module. High-quality sequences were aligned through the integrated Mafft aligner, while rooted and unrooted 16S phylogenetic trees were constructed using the QIIME2 phylogenetic module with the FastTree algorithm [14]. Taxonomic assignment of “representative” sequences was obtained by using the QIIME2 embedded Naïve Bayes fitted classifier, pre-trained on the most recent Greengenes reference database (ver. 13.8) [15]. The potential effects of read sampling depth on microbial diversity calculations were evaluated by the examination of the rarefaction curves, through the “diversity” QIIME module. Subsequently, several alpha diversity measures (Shannon’s diversity index [16], number of observed features, Faith’s phylogenetic diversity [17], Pielou’s evenness [18]) were computed, with the established sampling cutoff. Dissimilarity among samples (Jaccard [19], Bray–Curtis [20], unweighted UniFrac and weighted UniFrac distances [21]) was inspected with principal coordinate analysis (PCoA) plots using EMPeror web application [22] at https://view.qiime2.org (accessed on 23 March 2022). Group significance between alpha and beta diversity indexes was calculated with QIIME2 plugins using the Kruskal–Wallis test and permutational multivariate analysis of variance (PERMANOVA), respectively. Thus, the initial QIIME2-based procedure allows to perform basic metagenomics analyses, i.e., sequence quality check, feature table construction, and the determination of sample- and group-specific “global” properties (diversity measures).

Subsequently, “local” properties (differences in microbial composition) were investigated. We utilized tools that take care of the “compositional” nature of the microbiome dataset, in which the sum of feature tables (microbial taxa) within a sample is irrelevant and dependent on sequencing yield, while taxa-specific abundances are not independent estimates, being interacting/competing entities.

Differential abundance of the identified microbial taxa between the two groups at species, genus, and family levels was initially tested with ANCOM, “Analysis of Composition of Microbiomes” [23].

Before ANCOM analyses, contaminants (i.e., of mitochondrial/chloroplast origin) and ultra-rare, i.e., that appeared in less than five samples and those with less than 20 counts (across all samples) were removed. After observing ANCOM output, three additional compositional data analysis algorithms were used in order to obtain more consistent signals of a different microbial signature between groups (CD and HC). Specifically, Coda-lasso [24], Clr-lasso [25], and Selbal [26] methods were applied as described in https://malucalle.github.io/Microbiome-Variable-Selection/ (accessed on 23 March 2022), for a number of reasons: they were adequately conceived for sparse and compositional data (like microbiomes); their results could easily be compared; they were not computationally intensive; and are easily be implemented in R environment. Prediction of the samples’ genetic and functional content was carried out by utilizing the Picrust v2.3.0b software [27] on the filtered feature table and “representative” sequences deriving from the QIIME2 output. Differences in Picrust2 predicted pathway counts (“unstratified pathway abundances”, table where pathways are defined by their MetaCyc [28] identifiers) were evaluated by the analysis tool ALDEx2 [29], which was as also suggested within Picrust2 website (https://github.com/picrust/picrust2/wiki/PICRUSt2-Tutorial-(v2.4.2) (accessed on 23 March 2022). Briefly, the predicted pathway count table was split according to the two sample groups, and the ‘aldex’ command for pairwise analysis was run. From the ALDEx2 tabular output, we considered three progressive significance cutoffs (ALDEx2 “effect” parameter) of 0.5, 1, and 1.5 (absolute value), in order to detect differentially abundant pathways. Noteworthy, this functional analysis has a mere explorative purpose, given that it relies on predictions of gene/pathway counts. Analogously to microbial differential abundance analysis, these quantities were also treated as compositional data. Finally, microbial dysbiosis across CD samples was explored by implementing three different published methodologies on the QIIME2 filtered feature table and the HC group as a reference sample set.

The first method [30] is based on the comparison of sample-specific median Bray–Curtis distance (vs. each reference sample) with a cutoff distance (90% quantile of the distance distribution calculated within reference set). Basically, sample-to-sample Bray–Curtis dissimilarities were downloaded from the QIIME2 diversity output reports; median dissimilarity from each sample and the total pool of HC set were calculated. The 90% percentile value for Bray–Curtis dissimilarity distribution for the HC set was considered as a “dysbiotic” cut-off.

The second methodology [31] is based on the generation of a “Dissimilarity-Overlap Curve (DOC)” for the test sample and its comparison with the one obtained from the reference set. This method relies on the concept of “universal microbial dynamics”, in which samples with a high portion of shared taxa also have a comparable abundance profile for those taxa. Authors implemented their method to a series of simulated and real-world data, finding a distinct behavior of the curve among healthy (negative slope at high overlap values) and unhealthy/simulated data (a generally flat curve).

Third, the non-parametric outlier detection “CLOUD” test [32] was implemented in order to possibly identify CD samples that were significantly ecologically distant from a chosen subset of reference samples (test was repeated considering the 5, 15, and 30% of control samples as “reference”). This method is also based on the abovementioned Bray–Curtis dissimilarity matrix. It is based on the concept that a “normal” sample is approximately close to the chosen subset of HC set, while an outlier “dysbiotic” sample is largely distant from it. In fact, the CLOUD “r” statistics are a ratio between sample-to-reference subset neighborhood diameter and the average neighborhood diameter for HC set. Thus, “r = 3” for a CD sample would indicate that its neighborhood diameter is three times larger than the average diameter within the HC set.

R Package Microbiome [33] v.1.8.0 and Phyloseq [34] v1.30.0 were implemented for the differential abundance and dysbiosis analyses.

## 3. Results

A total of 46 CD patients aged 15–75 years from Andriulli et al. [16] were included in the study. Briefly, all CD had a biopsy-supported diagnosis of CD; negative serology for CD, namely anti-tissue transglutaminase 2 (tTG2), anti-gliadin (AGA) and anti-endomysial antibodies (EMA); and absence of gluten immunogenic peptides (GIP) and symptoms, and all subjects were in remission and on GFD for a minimum of 2 years and carriers of at least one of the DQ2-DQ8 alleles. The general characteristics of participants are given in Table 1. In addition, 30 specimens from HC were analyzed (13 females, mean age at recruitment 46 ± 7.98).

### 3.1. 16S rRNA V3-V4 Region Sequencing Results

After clustering, removal of chimeras, and filtering, the median sequencing output was 16841 reads with IQR 13210–23273 (Supplementary Data S1, Table S4).

Based on the rarefaction curve, the alpha and beta diversity metrics were calculated on a rarefied frequency-feature table with a minimum number of 10,000 sequences per sample. This sampling depth threshold determined the exclusion of only three samples (two CD patients and one HC) with sample identifiers “GF105” (total number of high quality reads = 9410), “GF040” (*n* = 9451), and “10C” (*n* = 7386), respectively. Details are shown in (Supplementary Data S1).

### 3.2. Fecal Microbiota Diversity Analysis

**Figure 1:**
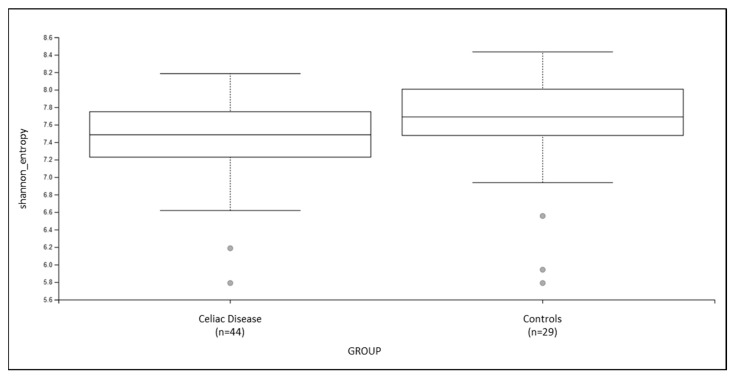
Boxplot for group-specific Shannon’s entropy distributions. See Table 2 and Supplementary Data S1 for details. Kruskal–Wallis test (all groups): *q* value = 0.07.

There was no difference in alpha diversity distribution comparison between the two groups (Figure 1 and Table 2).

**Figure 2:**
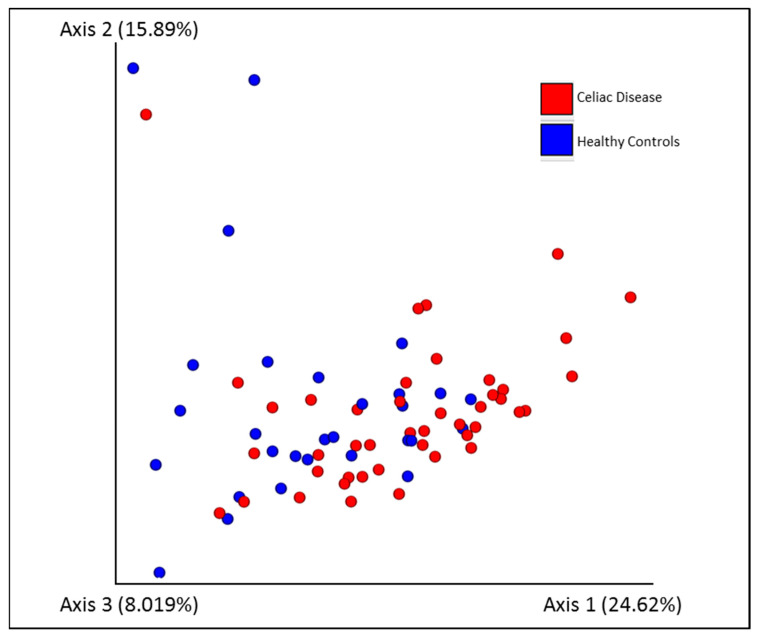
Principal coordinates analysis plot for HC (blue), and CD patient in GFD (red); distances across samples are calculated through Weighted Unifrac distance.

The CD cohort samples tend to cluster and be distant with respect to the HC, according to the PERMANOVA test, performed for the following beta diversity metrics: Jaccard similarity (Pairwise PERMANOVA *q*-value = 0.004, number of permutations = 999), Bray–Curtis dissimilarity (*q*-value = 0.005), Unweighted UniFrac (*q*-value = 0.06), and Weighted UniFrac (*q*-value = −0.001) dissimilarity (Figure 2 and Table 3).

These data suggested that microbiomes from CD patients in stringent GDF tend to resemble each other and differ to HC fecal microbiome, but their intrinsic diversity (alpha indexes) is in the range of the normal one (control set). Full results for diversity analysis are presented in Supplementary Data S1.

### 3.3. Microbial Signature Detection

From the QIIME2 ANCOM modules, run at Family (“L5”), Genus (“L6”), and Species (“L7”) levels, we showed that there are a couple of organisms more abundant in HC named *Bifidobacterium longum* (W statistics = 19) belonging to Bifidobacteriaceae family (W = 19) and *Coprococcus eutactus* (W = 15). In addition, differences in microbial composition between the two groups were also detected using Coda-lasso, Clr-lasso, and Selbal methods. We instructed the software to search for a maximum of 20 discriminant taxa (between CD and HC groups) to be selected across the whole feature table (shown in Supplementary Table S1, sheet 2). Global results from each tool are summarized in Supplementary Data S1, sheet 3. We observed that 22 taxa, which can be collapsed to 17 different taxonomies, were selected by at least one tool, as more abundant in controls. Fourteen taxa, which can be collapsed into 11 different taxonomic classifications, were enriched in CD patients rather than controls.

Taking into consideration the methodological heterogeneity, we tried to focus only on the most consistent taxon among at least one of the microbial classifiers used (Coda-lasso, Clr-lasso and Selbal) and the QIIME2 taxonomic classification, thus finding *Bifidobacterium longum* and *Coprococcus eutactus* more abundant in HC samples. Furthermore, we assessed that *Coprococcus catus* and *Blautia* genus, both belonging to Lachnospiraceae family, were also over-represented in HC, whereas *Bacteroides* genus was found to be more abundant in CD (Supplementary Table S1).

### 3.4. Differential Analysis of Predicted Functional Content

A total of 1561 gene functions (defined by Enzyme Commission identifiers) and 312 metabolic pathways (defined by MetaCyc identifiers) were inferred using the PICRUSt pipeline across the 76 samples.

Given the PICRUSt “unstratified” (i.e., quantities are not stratified per sample) pathway counts table, we identified only two mildly differentially abundant pathways with an ALDEx “effect” cutoff of 0.5 (the most relaxed threshold). Both pathways, namely tetrapyrrole biosynthesis I, from glutamate (MetaCyc identifier: PWY-5188, ALDEx2 effect = −0.58), and tetrapyrrole biosynthesis I from glycine (PWY-5189, ALDEx2 effect = −0.64) had a slightly greater abundance in the control group (Supplementary Table S2, sheet 3). Tetrapyrroles are organic molecules that contain four-membered heterocyclics (pyrrole) rings. One of them is cobalamin or vitamin B12, a cobalt-containing modified *tetrapyrrole.* In CD patients, the GFD reduces the absorption of various deficiencies of nutrients, dietary minerals, and vitamins, such as vitamin B12.

### 3.5. Dysbiosis Analysis of CD Patients vs. Controls

Microbial profiles of the CD patients were consistently inferred as “non-dysbiotic” according to the three methodologies (see Methods). Only one patient, the “GF014”, was found to be moderately dysbiotic according to the CLOUD test [32], having an estimated “r stat” of 1.5 (i.e., its neighborhood diameter is 1.5 larger than the average neighborhood diameter calculated in the control set) and a *p*-value < 0.05. Interestingly, according to the first described method [30], this sample had a median Bray–Curtis distance (vs. control set) very close to the computed dysbiotic cutoff (BC distance = 0.90485).

Regarding the “Dissimilarity-Overlap” test [31], regression curves that are compatible with an “healthy” microbial dynamics were obtained for both CD and HC sample sets. For both sets, a negative correlation (Spearman Correlation Coefficients of −0.68 and −0.79 for CD and HC curves, respectively) for dissimilarity vs. overlap distributions was observed in the region of high overlap. Details are shown in the Supplementary Data S2.

## 4. Discussion

Recent studies [5,6,7,35] have not well elucidated if the altered microbiota composition is involved in CD pathogenesis or if these are secondary effects of disease pathology. Worldwide, the common efficient treatment for CD is lifelong adherence to a strict GFD that can fully restore mucosal morphology, but on the other hand, it is known that the adherence to a GFD may be associated with digestive problems due to insufficient intake of dietary fiber, vitamins, and other nutrients, thus affecting microbial fiber-fermenting species and colonic production of short-chain fatty acids (SCFAs).

In the current study, we analyzed the fecal microbial composition of subjects with a biopsy-supported diagnosis of CD, carriers of at least one of the human leukocyte antigen DQ2 and DQ8 risk alleles, negative serology for CD, absence of GIP in stool samples, absence of symptoms, and on a GFD for a minimum of 2 years. The data on the microbiota of celiac people and on the changes/differences occurring between celiac on a GFD and healthy people are still contradictory, depending on several factors (the duration of GFD, the strong inter-individual variability, different protocols etc.); however, in studies similar to our research, there is evidence on a partial restoration of gut microbiota, although with lower levels of beneficial bacteria [36].

Our analysis revealed that CD patients in GFD had an almost stable (i.e., non-dysbiotic) microbiome at least at crude alpha diversity indexes (i.e., species richness and evenness, observed features, and phylogenetic diversity were similar among CD samples in GFD compared to those from HC). Data were also confirmed when three different methodologies for depicting dysbiosis were applied to our cohort. Instead, a significant effect was observed in all beta diversity metrics analyzed as compared to those from HC, showing a dissimilarity between the two microbial communities.

More importantly, subsequent analysis by means of compositional data analysis algorithms (ANCOM, Coda-lasso, Crl-lasso, and Selbal) showed that there were some bacterial species that were less abundant in CD patients, while *Bacteroides* taxa resulted in more abundance.

Two of the species resulted in less abundance in CD patients, namely *Bifidobacterium longum* and *Coprococcus eutactus,* and were in concordance among most of the classifiers used. It is well known that the prevalence of *Bifidobacterium* was significantly higher in controls than in the active CD group and that bifidobacteria may play an important role in the digestion process of intact gluten proteins and toxic gluten-derived peptides. In particular, *Bifidobacterium longum* showed lower numbers of taxon in CD patients, especially between active CD disease and HC [37]. In a further study, McCarville JL et al. [38] demonstrated that *Bifidobacterium longum* NCC2705 attenuates gliadin-induced immunopathology and impacts intestinal microbial composition in gliadin-sensitized NOD/DQ8 mice.

Our analysis highlighted that CD patients in GFD had a decreased level of three taxa belonging to Lachnospiraceae family, two of them at the *Coprococcus* genus, namely *Coprococcus eutactus* and *Coprococcus catus,* and the third at the *Blautia* genus.

Although Lachnospiraceae are among the main producers of SCFA, some taxa of Lachnospiraceae are also associated with different intra- and extraintestinal diseases [39], and it has been determined that pseudocereals decreased their levels [40].

A reduced absolute and relative abundance of *Bifidobacterium* spp., of a couple of species belonging to Lachnospiraceae family and one from *Blautia* were the most important effects of a low-gluten diet in non-celiac healthy adults driven by qualitative changes in dietary fibers [41].

Otherwise, we observed a significant difference in *Bacteroides* genus that resulted enriched in our well-characterized cohort of CD in GFD. This abundance is in accordance with previous literature aimed at exploring the microbial composition from both fecal and biopsies pinched from duodenal mucosa of CD patients. In particular, microbiota studies, focused on the contribution of the HLA alleles in celiac patients, have revealed an increase of *Bacteroides* especially in those carriers of the risk alleles [42,43]. In addition, *Bacteroides* were more abundant in feces and biopsies of CD patients than in controls regardless of the stage of the disease [44]. These data were also confirmed in a study performed on duodenal biopsies [45].

At the application of ALDEx2 differential abundance analysis with assessed effect sizes and stringent PICRUSt2-predicted metabolic pathways, we pinpointed a couple of pathways related to microbial composition with a significant reduction in the CD group, namely tetrapyrrole biosynthesis I from glutamate (PWY-5188), and tetrapyrrole biosynthesis I from glycine (PWY-5189).

Guidelines from the American College of Gastroenterology [46] and from the United Kingdom National Institute for Health [47] strongly suggest to investigate, at the time of CD diagnosis, also for vitamins, minerals, and nutritional deficiencies, such as calcium, magnesium, zinc, vitamin D, folic acid, and vitamin B12. Numerous studies have shown that circulating levels of this vitamin are inadequate in up to 40% of patients with CD independently from the diet [48]. In our cohort, 4 (8.7%) of the CD patients had a vitamin B12 level below the range (193–986 pg/mL) and the other four subjects (8.7%) between 193 and 250 pg/mL, respectively, which are the cutoff levels in adults for deficiency and depletion in the vitamin [49] (data not shown).

## 5. Conclusions

In the present study, we aimed to dissect the microbial composition of CD patients in GFD as compared to that from healthy controls. We pinpointed that CD patients in GFD had a quite stable microbial composition for the crude alpha diversity metrics, showing us that their microbiota had a phylogenetical distance, a number of OTU, and an evenness not different as compared to HC.

We found significant differences in beta diversity and in the abundance of certain taxa between subjects with inactive CD in GFD and controls. Our work emphasizes the commonalities among many published studies on CD and someone might conclude that a decrease in Bifidobacteria and an increase in *Bacteroides* seem to be a somewhat common denominator across the studies, both on feces and on mucosal biopsies. In addition, the negative effect of the tetrapyrrole biosynthesis pathways and the identified inadequate level of Vitamin B12 in the patient cohort could reflect an effect of nutrient deficiency on malabsorption of vitamins that may influence the CD pathogenesis. The suppression of taxa-producing SCFAs, those with an important role in the digestion process of intact gluten proteins and the inadequate levels of vitamins, could advise the development of new gluten-free products by modulating the gut microbiota through diet, supplements of vitamins, and the addition of specific prebiotics.

## Figures and Tables

**Table 1:** Characteristics of randomized patients with celiac disease.

N of patients	46
Female	28
Age in years, mean ± SD	38.6 ± 18.2
Measurement	
Height, means ± SD in cm	168.6 ± 9.5
BMI mean ± SD	22.7 ± 3.5
Duration of GFD in years mean ± SD	11.1 ± 7.4
2–3.9	4
4–5.9	10
6–7.9	7
>8	25
HLA	
DQ2	40
DQ8	2
DQ2–DQ8	4

**Table 2:** Summary of pairwise group comparisons for four alpha diversity indexes. Benjamini and Hochberg corrected *p*-values (*q*-values) for Kruskal–Wallis tests are shown.

	Healthy Control vs. Celiac Disease
Shannon’s Entropy	0.07
Pielou’s evenness	0.75
Number of Observed Features	0.09
Faith’s Phylogenetic Distance	0.34

**Table 3:** Summary of pairwise group comparisons for beta diversity measures. Benjamini and Hochberg corrected *p*-values (*q*-values) for PERMANOVA tests are shown.

	Healthy Control vs. Celiac Disease
Jaccard similarity	0.004
Bray-Curtis dissimilarity	0.005
Unweighted UniFrac dissimilarity	0.06
Weighted UniFrac dissimilarity	0.001

## Data Availability

The data presented in this study are all available in the Supplementary Files.

## Supplementary Materials

The following supporting information can be downloaded at: https://www.mdpi.com/article/10.3390/nu14122452/s1, Table S1: microbial differential abundance analysis re-sults by Coda_lasso, Clr_lasso, and Selbal methods; Table S2: pathway prediction and associated differential abundance measurement by Picrust2 and ALDEx2 tools; Table S3: Se-quencing and alignment statistics; Table S4. Feature distribution per sample (“per sample” col-umn) and global feature distribution (“per feature”); Table S5: Median Bray-Curtis (“BC”) dis-tances for CD samples with respect to the HC control set; Table S6. Results for the CLOUD test; Figure S1: Pielou’s evenness; Figure S2: Faith’s Phylogenetic Distance; Figure S3: Number of Observed Features; Figure S4: Shannon entropy; Figure S5: Bray-Curtis; Figure S6: Jaccard; Figure S7: Unweighted Unifrac; Figure S8: Weighted Unifrac; Figure S9: Results for species-collapsed features (n = 118); Figure S10: Density plot of BC distances in the HC (‘Control’) and CD (‘Basal’) sample sets; Figure S11: Scatterplots for the Dissimilarity vs. Overlap estimations within the CD (‘Basal’) and HC (‘Control’) sample sets.
