# Supplementary material for: Adherence to Gluten-Free Diet Restores Alpha Diversity in Celiac People but the Microbiome Composition Is Different to Healthy People
Source: Nutrients. 2022 Jun 14;14(12):2452. doi: 10.3390/nu14122452 (PMC9228530; doi:10.3390/nu14122452)
Supplement: Supplementary file 1 [file nutrients-14-02452-s001.zip › Supplementary_Data S1.pdf]

Supplementary Data S1

Table S3. Sequencing and alignment statistics. Per- sample Sequencing statistics. First column: sample identifier; columns 2 – 8: absolute counts and percentages for surviving reads after QIIME2 DADA2 implementation.

Sample ID	# reads	# filtered	% filtered	#denoised	#merged	% input merged	# non-chimeric	% non-chimeric
**GF001**	231491	185240	80.02	180450	162434	70.17	29364	12.68
**GF002**	116476	100044	85.89	96985	81764	70.2	14184	12.18
**GF003**	161391	92846	57.53	88966	72289	44.79	14854	9.2
**GF005**	114766	68667	59.83	66672	57001	49.67	12334	10.75
**GF006**	562794	466442	82.88	462373	448227	79.64	80677	14.34
**GF007**	326515	244888	75	237602	201279	61.64	34224	10.48
**GF008**	397607	324455	81.6	317400	283517	71.31	39431	9.92
**GF009**	333530	260659	78.15	254086	227032	68.07	35659	10.69
**GF011**	170922	109083	63.82	105968	89256	52.22	16354	9.57
**GF013**	237362	152243	64.14	149908	133753	56.35	19783	8.33
**GF014**	261668	188878	72.18	184449	165642	63.3	26937	10.29
**GF015**	230576	165929	71.96	159360	133221	57.78	23402	10.15
**GF016**	193680	152264	78.62	148534	129415	66.82	20055	10.35
**GF017**	114634	91437	79.76	89322	78433	68.42	14261	12.44
**GF018**	308471	206063	66.8	201720	179528	58.2	24067	7.8
**GF019**	115357	70592	61.19	68056	57458	49.81	11893	10.31
**GF020**	160711	131920	82.09	127230	107228	66.72	15528	9.66
**GF023**	529083	396583	74.96	386683	340346	64.33	46300	8.75
**GF024**	230965	175989	76.2	172092	152201	65.9	19051	8.25
**GF025**	162860	100236	61.55	98024	87500	53.73	17937	11.01
**GF026**	218906	182049	83.16	179875	167673	76.6	18952	8.66
**GF027**	143433	113873	79.39	110143	92647	64.59	15974	11.14
**GF028**	94414	61405	65.04	60290	54662	57.9	11210	11.87
**GF029**	132150	85591	64.77	83411	69582	52.65	13896	10.52
**GF031**	179228	114629	63.96	110840	92695	51.72	18549	10.35
**GF032**	132171	112612	85.2	108874	89634	67.82	10717	8.11
**GF033**	100392	60239	60	58222	47369	47.18	12359	12.31
**GF037**	132711	114604	86.36	111251	94933	71.53	11830	8.91
**GF040**	76093	56656	74.46	54048	44317	58.24	9451	12.42
**GF041**	148500	114001	76.77	110058	90977	61.26	12654	8.52
**GF42**	152338	66761	43.82	64666	56515	37.1	10246	6.73
**GF043**	118209	91221	77.17	88029	74415	62.95	12185	10.31
**GF044**	122886	99292	80.8	96877	84394	68.68	14150	11.51
**GF048**	163577	130821	79.98	127298	110997	67.86	13179	8.06
**GF053**	277318	226460	81.66	222631	200009	72.12	21170	7.63
**GF054**	150487	123219	81.88	119727	105211	69.91	15823	10.51
**GF060**	221267	159046	71.88	156271	139574	63.08	20261	9.16
**GF061**	108981	80233	73.62	77302	63396	58.17	12543	11.51
**GF062**	124553	102948	82.65	99564	83780	67.26	11971	9.61
**GF063**	251719	172639	68.58	168425	143991	57.2	19272	7.66

**GF100**	193574	137465	71.01	134793	119066	61.51	20845	10.77
**GF104**	242022	204730	84.59	200227	172240	71.17	22828	9.43
**GF105**	85311	66930	78.45	64861	54781	64.21	9410	11.03
**GF106**	293200	229475	78.27	225622	200511	68.39	26301	8.97
**GF108**	166732	138265	82.93	135627	122736	73.61	17572	10.54
**GF111**	367450	286188	77.88	278236	236417	64.34	27837	7.58
**10C**	115538	50658	43.85	47763	36555	31.64	7386	6.39
**11C**	333911	148888	44.59	143856	123882	37.1	21454	6.43
**12C**	161464	75287	46.63	74187	68880	42.66	10316	6.39
**1C**	221871	87400	39.39	85387	74812	33.72	11885	5.36
**2C**	409927	193644	47.24	191101	180275	43.98	31608	7.71
**4C**	402377	196069	48.73	190090	164806	40.96	26791	6.66
**5C**	292050	120821	41.37	116659	96068	32.89	14975	5.13
**6C**	276142	111430	40.35	108799	98575	35.7	18544	6.72
**7C**	324335	139489	43.01	135453	117216	36.14	16909	5.21
**8C**	541443	256702	47.41	249813	216613	40.01	30584	5.65
**CON652SGR-06-09-2021**	193330	130727	67.62	123196	93798	48.52	15729	8.14
**CON653SGR-20-10-2021**	218667	153358	70.13	147132	118670	54.27	23230	10.62
**CON658SGR-06-09-2021**	183060	135549	74.05	128358	102087	55.77	14598	7.97
**CON659SGR-06-09-2021**	238868	188386	78.87	181525	152661	63.91	17866	7.48
**CON660SGR-08-09-2021**	159146	115942	72.85	112243	90153	56.65	13693	8.6
**CON661SGR-06-10-2021**	208649	133871	64.16	128914	105663	50.64	21426	10.27
**CON662SGR-06-10-2021**	109757	83858	76.4	79721	60245	54.89	13221	12.05
**CON663SGR-06-10-2021**	268645	191306	71.21	185499	158310	58.93	18426	6.86
**CON664SGR-07-10-2021**	122688	75899	61.86	73489	62444	50.9	13775	11.23
**CON665SGR-08-10-2021**	172435	129878	75.32	125546	103873	60.24	16773	9.73
**CON666SGR-13-10-2021**	167347	115243	68.86	112648	100970	60.34	15717	9.39
**CON667SGR-14-10-2021**	124097	83706	67.45	81486	68817	55.45	11746	9.47
**CON668SGR-30-12-2021**	246404	182035	73.88	179215	165255	67.07	23999	9.74
**CON669SGR-30-12-2021**	202119	159507	78.92	157157	144839	71.66	29815	14.75
**CON670SGR-30-12-2021**	260862	198693	76.17	195730	180693	69.27	25709	9.86
**CON671SGR-30-12-2021**	302717	205184	67.78	201414	181428	59.93	30546	10.09
**D01SGR-18-03-2021**	295998	218052	73.67	211333	184354	62.28	27848	9.41
**D02SGR-22-04-2021**	145683	101912	69.95	98894	84615	58.08	15908	10.92
**D03SGR-22-04-2021**	197762	123744	62.57	118392	92796	46.92	15489	7.83
**D04SGR-23-06-2021**	122531	89905	73.37	86722	72313	59.02	12583	10.27

Table S4. Feature distribution per sample ("per sample" column) and global feature distribution ("per feature"). The number of high quality sequence count ranges from 7386 to 80677, while feature abundance ranges from 1 to 24426 counts.

	Per sample	***Per feature***
Min. frequency	7386	1
1st quartile	13210	2
Median frequency	16841	4
3rd quartile	23273	18
Max. frequency	80677	24426
Mean frequency	19684	89

Alpha diversity analysis.

We investigated Alpha diversity by calculating four different measures: Pielou's evenness (1.4.1), Faith's Phylogenetic Distance (1.4.2), Number of Observed Features (1.4.3) and Shannon's entropy (1.4.4). For each index, the following outcomes are provided: boxplot for group-specific index distribution (x-axis: 2 groups, "Celiac Disease" and "Healthy Controls"; y-axis: alpha-diversity index); Kruskal-Wallis test results (H statistics; p-value) for the global comparison of group-specific distribution; Kruskal-Wallis test results for pairwise group comparisons. Boxplot 1.4.4 is also reported in the main text, Figure 1. Analyses were performed through the "QIIME2 Diversity" module, using "10296" as cut-off for reads sampling depth (see main text for further details).

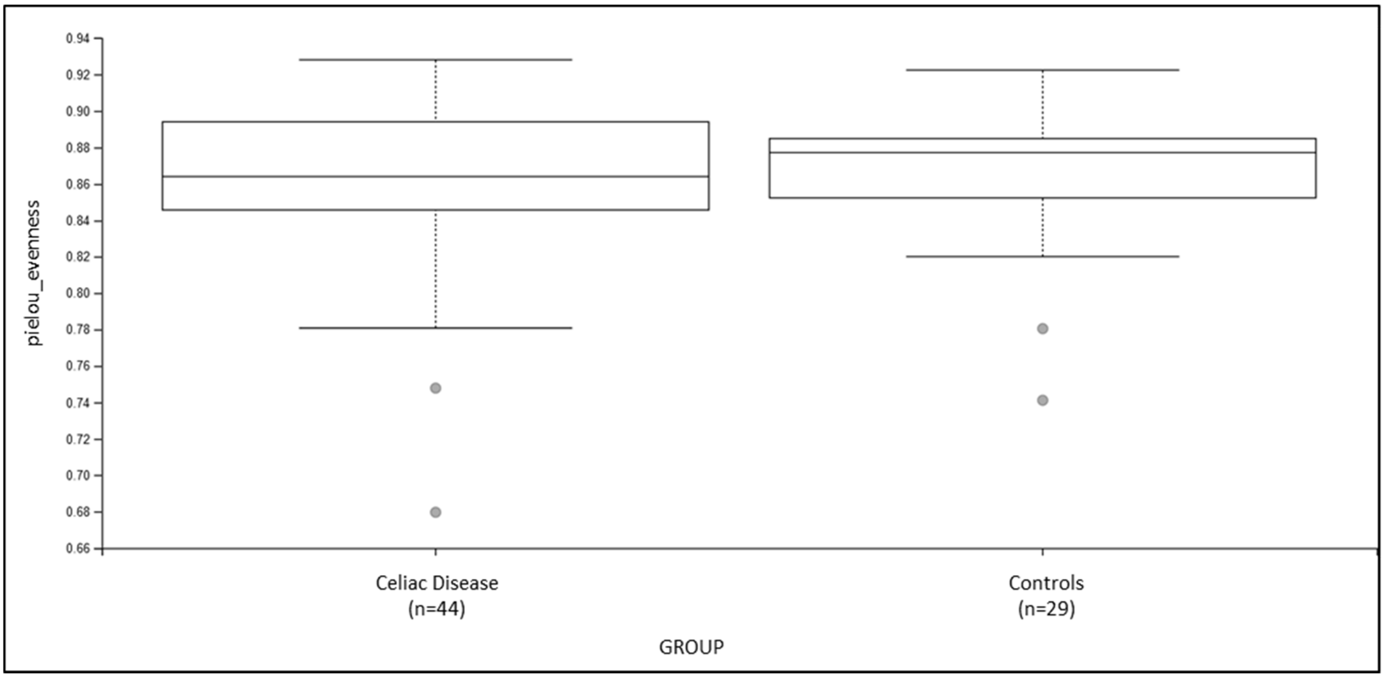

Kruskal-Wallis test (pairwise):

Group 1	Group 2	H	p-value	q-value
Celiac Disease	Healthy_Controls	0.0996	0.75	0.75

Figure S1. Pielou's evenness.

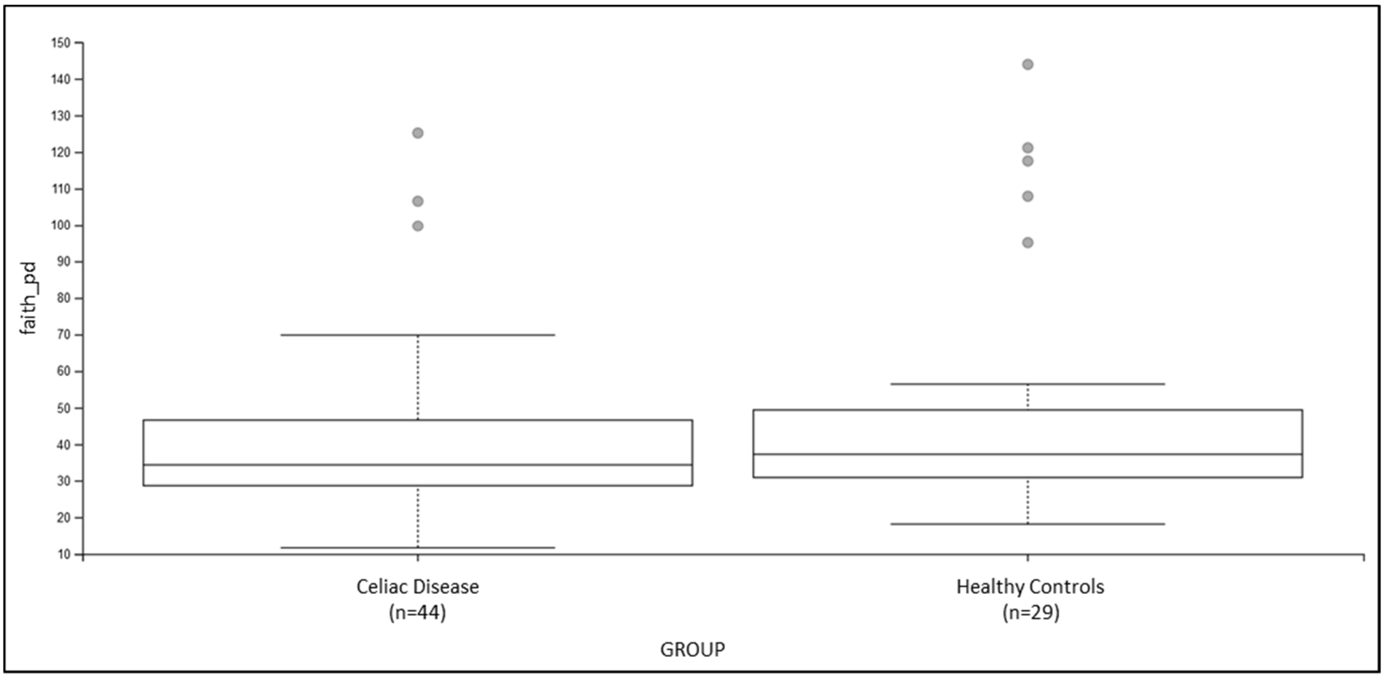

Kruskal-Wallis test (pairwise):

**Group 1**	**Group 2**	**H**	**p-value**	**q-value**
Celiac Disease	Healthy_Controls	0.897	0.34	0.34

Figure S2. Faith's Phylogenetic Distance.

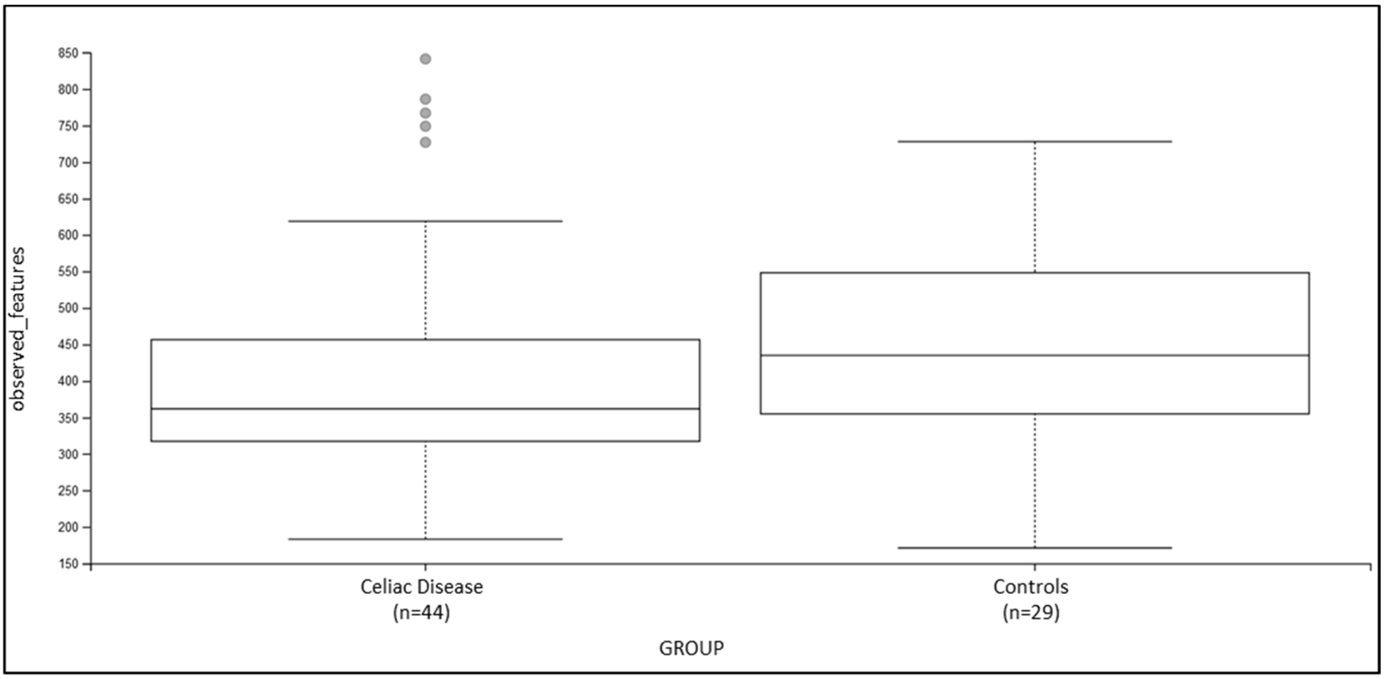

Kruskal-Wallis test (pairwise):

Group 1	Group 2	H	p-value	q-value
Celiac Disease	Healthy_Controls	2.86	0.091	0.091

Figure S3. Number of Observed Features.

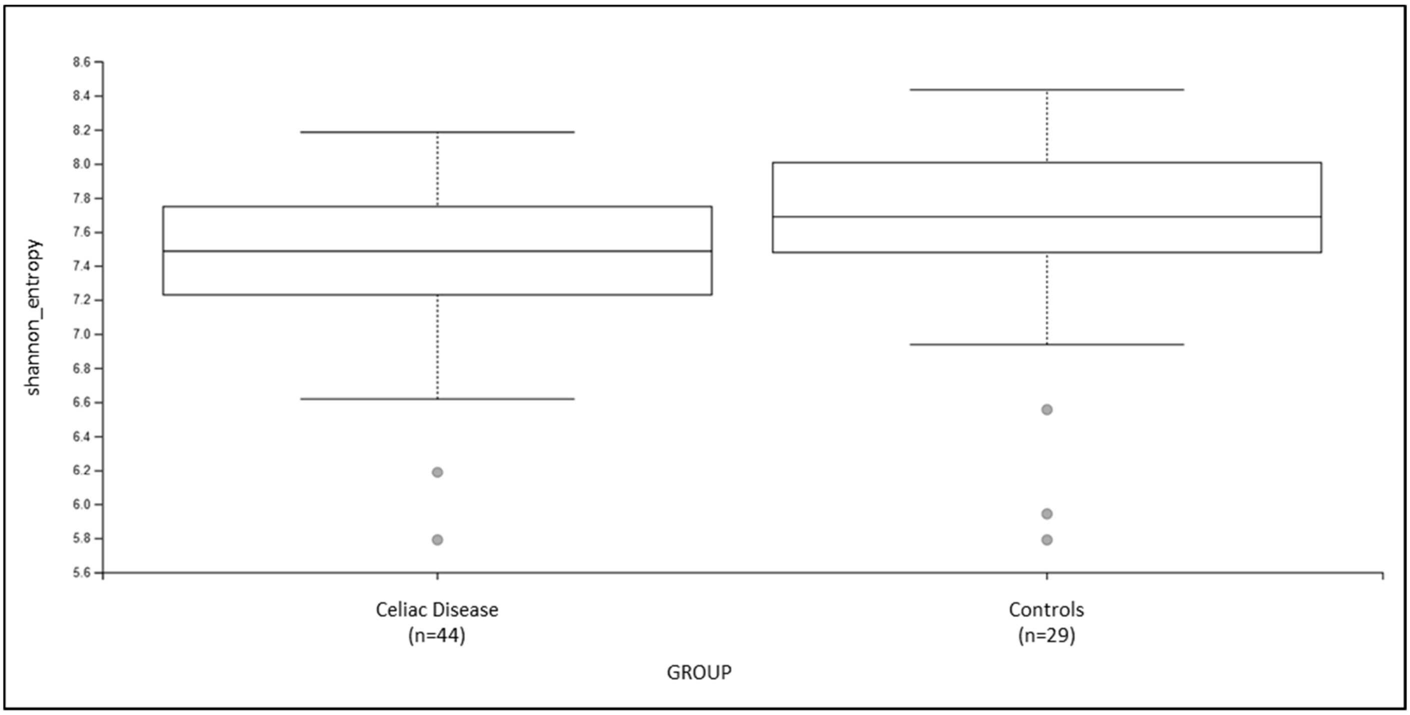

Kruskal-Wallis test (pairwise):

Group 1	Group 2	H	p-value	q-value
Celiac Disease	Healthy_Controls	3.212	0.073	0.073

Figure S4. Shannon entropy.

Beta diversity analysis.

Diversity between the two groups has been evaluated by considering four popular dissimilarity/distance methods: Bray-Curtis (1.5.1), Jaccard (1.5.2), Unweighted and Weighted Unifrac (1.5.3 and 1.5.4). For each method, the following outcomes are provided: a screenshot of three-dimensional Principal Coordinate Analysis ("3D-PCoA") plot; a table summarizing PERMANOVA test results for pairwise group comparisons. PCoA plots have been obtained by using the Emperor web-application within QIIME2 website (https://view.qiime2.org/); red dots indicate "Celiac Disease" samples and blue: "Healthy Controls. Furthermore, each table show details on comparing groups and their size, number of permutations, pseudo-F statistics, p-values and Benjamini-Hochberg adjusted p-value. PERMANOVA verifies the hypothesis that distances among samples with one class group differ from distances of these samples from samples of other groups. PCoA plot obtained by using Unweighted Unifrac distance is also reported in Main Text, as Figure 2.

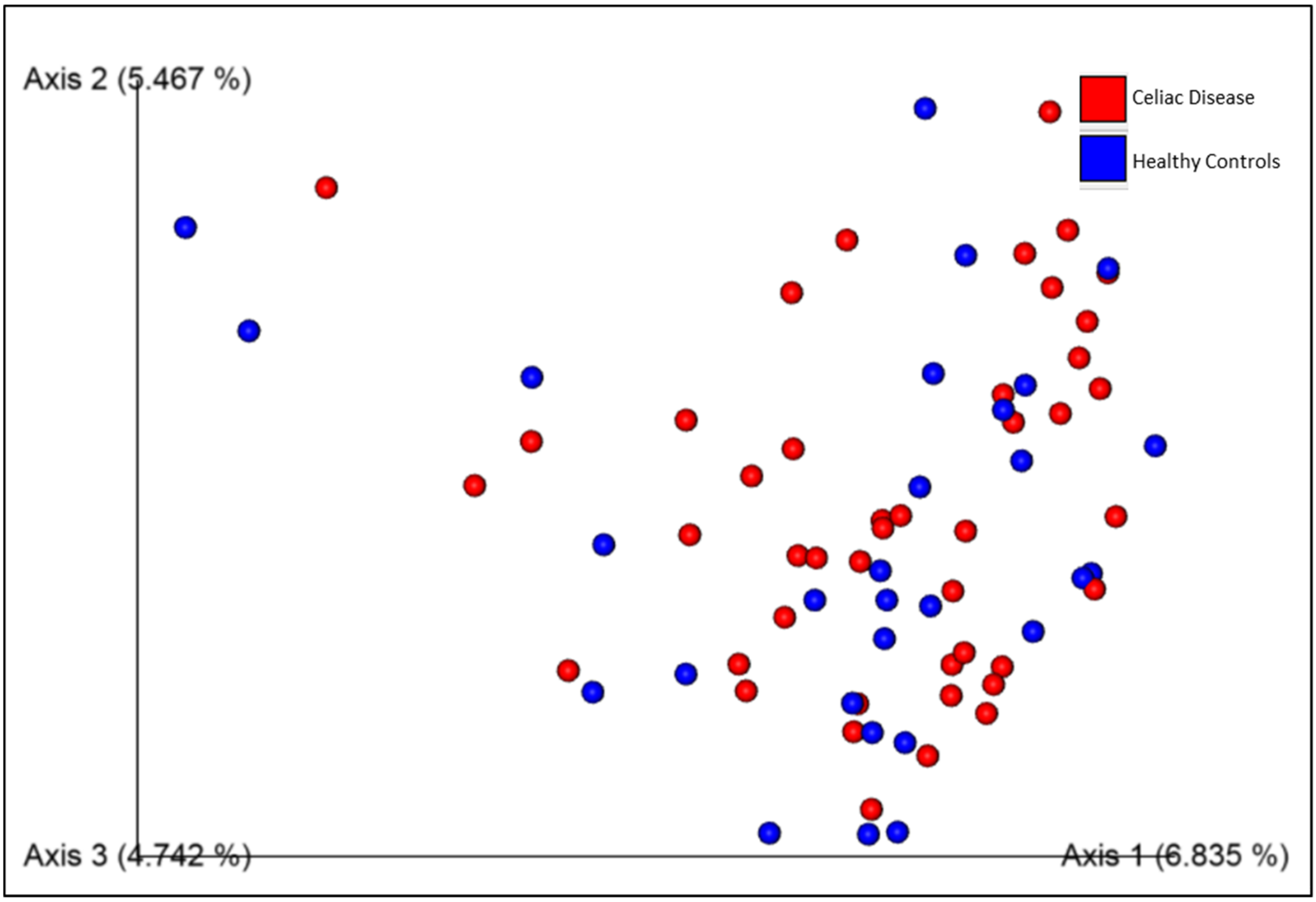

Kruskal-Wallis test (pairwise)

Group 1	Group 2	Sample size	Permutations	pseudo-F	p-value	q-value
Celiac Disease	Healthy Controls	73	999	1.561	0.005	0.005

Figure S5. Bray-Curtis.

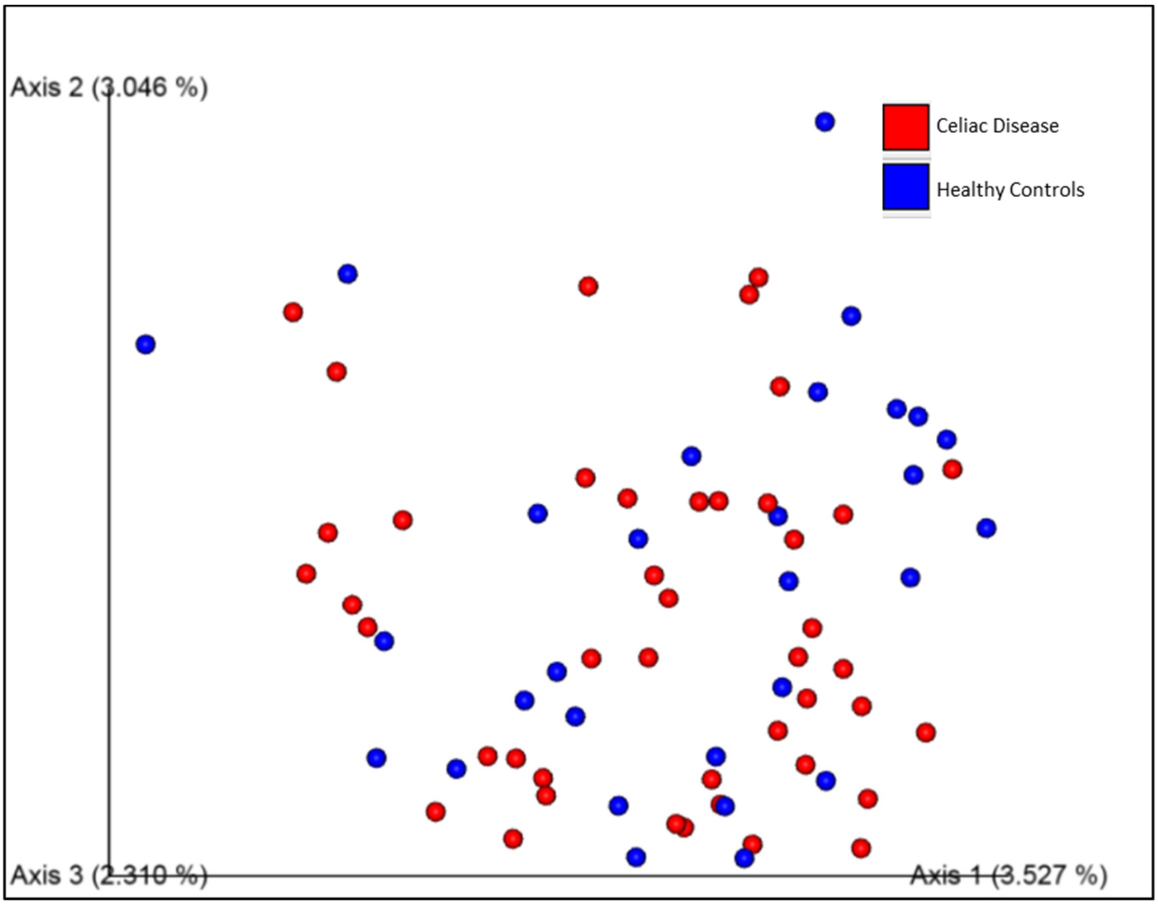

Kruskal-Wallis test (pairwise)

Group 1	Group 2	Sample size	Permutations	pseudo-F	p-value	q-value
Celiac Disease	Healthy Controls	73	999	1.190	0.004	0.004

Figure S6. Jaccard.

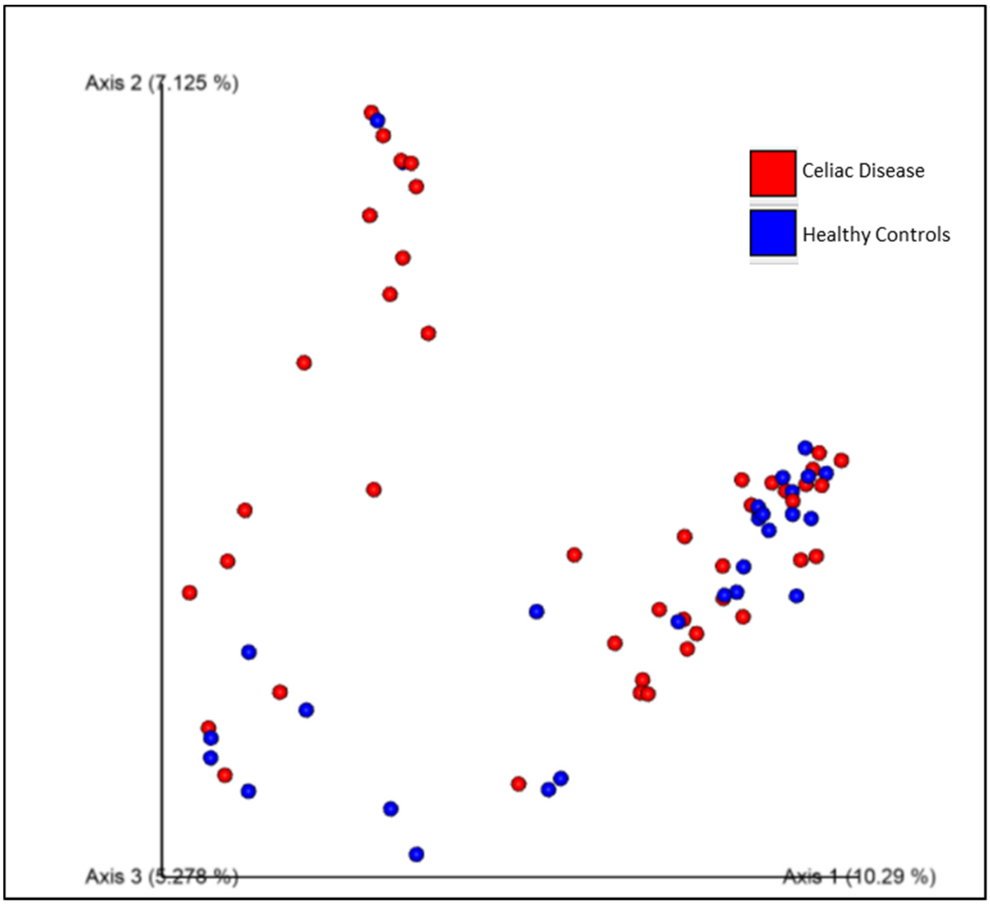

Kruskal-Wallis test (pairwise)

Group 1	Group 2	Sample size	Permutations	pseudo-F	p-value	q-value
Celiac Disease	Healthy Controls	73	999	1.286	0.064	0.064

Figure S7. Unweighted Unifrac.

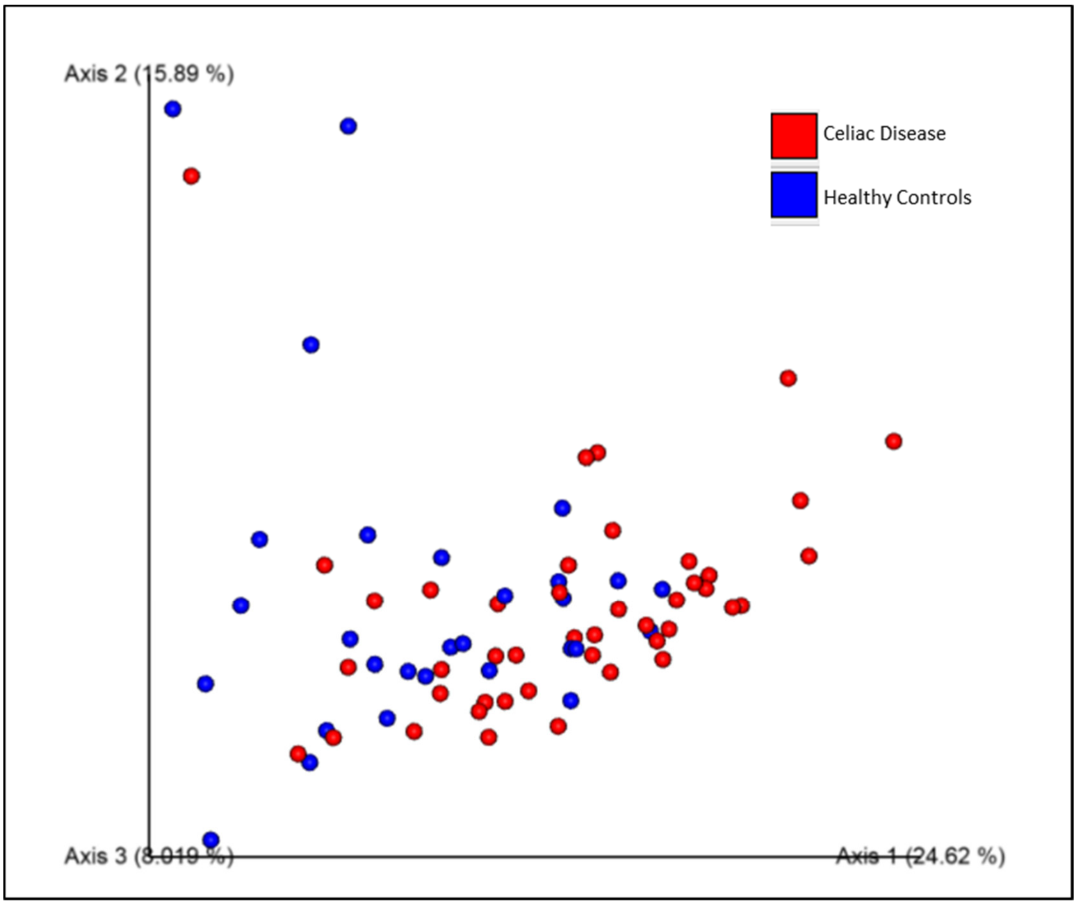

Kruskal-Wallis test (pairwise)

Group 1	Group 2	Sample size	Permutations	pseudo-F	p-value	q-value
Celiac Disease	Healthy Controls	73	999	4.576	0.001	0.001

Figure S8. Weighted Unifrac.

ANCOM

QIIME2 ANCOM module was used to infer microbial genera or species that are differentially abundant across sample groups. Rare features and mitochondrial/plastid sequences were removed from the feature table (see Methods). Thus, features were collapsed according to QIIME2 taxonomical classification (based on GreenGenes resource) into "species-collapsed" ("level 7") features, for a total of 118 features. ANCOM Volcano Plot and statistical results table are provided.

Significant features are placed on the top-right corner of the Volcano Plot; the first table shows significant features together with the corresponding W statistics, i.e., the number of sub-hypotheses that have passed for a certain feature (ANCOM compares pairs of feature relative abundances). The second table shows the percentile abundance of relevant features across the sample groups. For example, a value of 100 at 50% percentile indicates that the detected feature has a maximum sequence count of 100 in the 50% of samples of an investigated group.

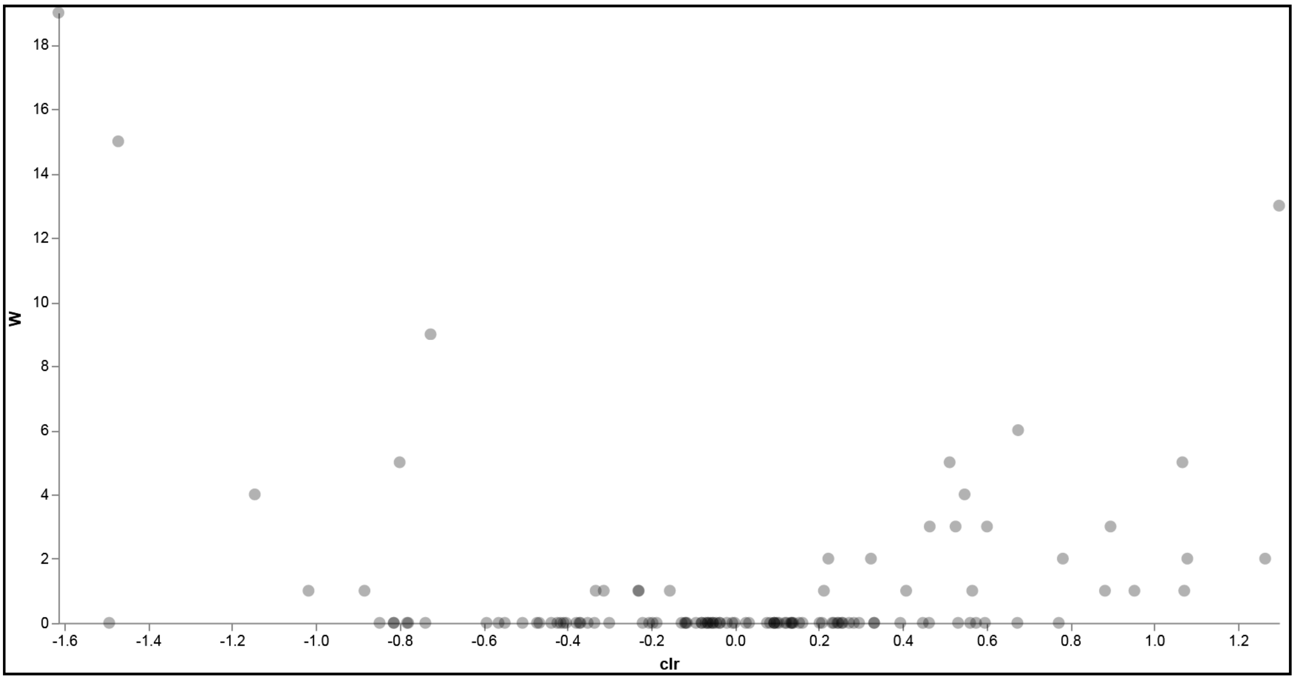

Feature	W
[A] k__Bacteria;p__Actinobacteria;c__Actinobacteria;o__Bifidobacteriales;f__Bifidobacteriaceae;g__Bifidobacterium;s__longum	19
[B] k__Bacteria;p__Firmicutes;c__Clostridia;o__Clostridiales;f__Lachnospiraceae;g__Coprococcus;s__eutactus	15
[C] k__Bacteria;p__Firmicutes;c__Clostridia;o__Clostridiales;f__Ruminococcaceae;g__Anaerotruncus;s__	13

Percentile	Group	[A]	[B]	[C]
	W	19	15	13
0.0	Celiac Disease	1.0	1.0	1.0
25.0	Celiac Disease	1.0	1.0	1.0
50.0	Celiac Disease	1.0	1.0	1.0
75.0	Celiac Disease	34.25	1.0	41.75
100.0	Celiac Disease	165.0	146.0	129.0
0.0	Healthy Controls	1.0	1.0	1.0
25.0	Healthy Controls	3.0	1.0	1.0
50.0	Healthy Controls	47.0	1.0	1.0
75.0	Healthy Controls	75.75	107.75	1.0
100.0	Healthy Controls	482.0	243.0	400.0

Figure S9. Results for species-collapsed features (*n* = 118).
