# Supplementary material for: Adherence to Gluten-Free Diet Restores Alpha Diversity in Celiac People but the Microbiome Composition Is Different to Healthy People
Source: Nutrients. 2022 Jun 14;14(12):2452. doi: 10.3390/nu14122452 (PMC9228530; doi:10.3390/nu14122452)
Supplement: Supplementary file 1 [file nutrients-14-02452-s001.zip › Supplementary_Data S2.pdf]

Supplementary Data S2

Table S5. Median Bray-Curtis ("BC") distances for CD samples with respect to the HC control set. Quantiles for control set "within" distances: 0.55 (0%); 0.77 (25%); 0.81 (50%); 0.86 (75%); 0.98 (100%). "Dysbiotic" 90% cut-off distance: 0.9094. BC pairwise dissimilarities were calculated through QIIME2, while dysbiotic cut-off and median distances were computed according from Lloyd-Pryce et al. 2019.

Patient ID	Median distance
**GF006**	0.7898
**GF007**	0.8031
**GF008**	0.8029
**GF009**	0.8033
**GF014**	0.8837
**GF015**	0.8318
**GF023**	0.8256
**GF003**	0.8328
**GF005**	0.791
**GF011**	0.7757
**GF013**	0.7919
**GF018**	0.7768
**GF019**	0.8171
**GF025**	0.7793
**GF028**	0.7979
**GF029**	0.7897
**GF031**	0.7991
**GF033**	0.7983
**GF017**	0.785
**GF024**	0.7742
**GF026**	0.8193
**GF041**	0.8153
**GF043**	0.7827
**GF054**	0.8022
**GF060**	0.8118
**GF061**	0.7711
**GF063**	0.8234
**GF100**	0.7632
**GF106**	0.8411
**GF108**	0.8246
**GF111**	0.7903
**GF002**	0.8147
**GF016**	0.7487
**GF020**	0.819
**GF027**	0.8165
**GF032**	0.7852
**GF037**	0.8247
**GF044**	0.807
**GF048**	0.7748
**GF053**	0.8675
**GF062**	0.8616
**GF104**	0.8005
**GF42**	0.7978
**GF001**	0.8099

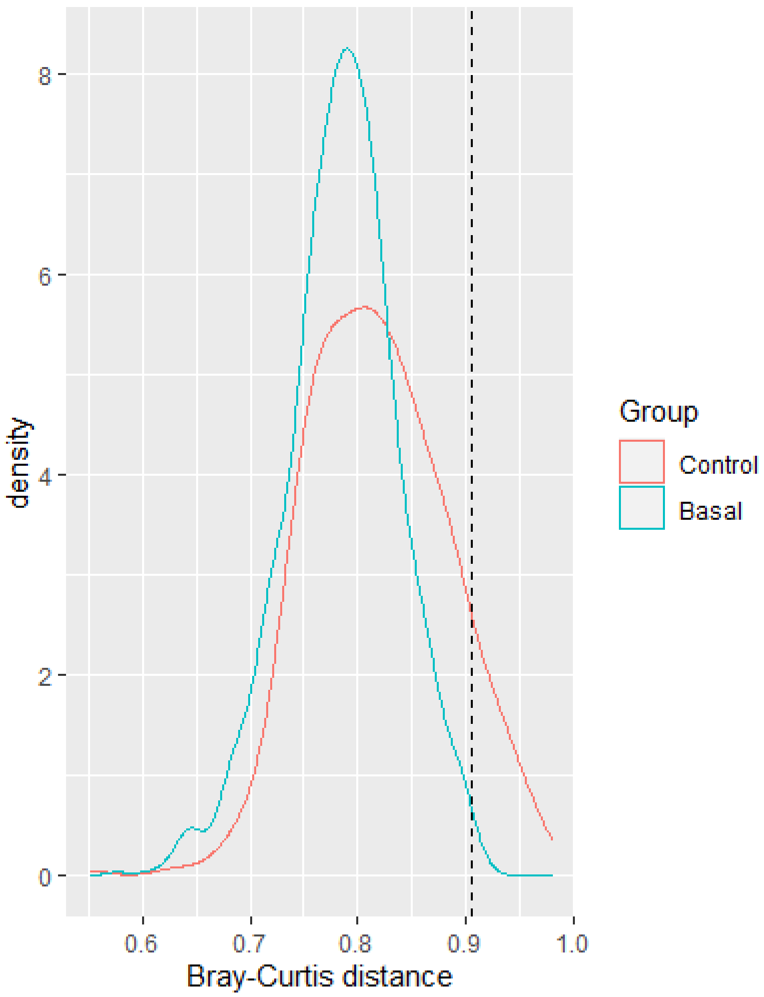

Figure S10. Density plot of BC distances in the HC ('Control') and CD ('Basal') sample sets. Dashed vertical line represents the "dysbiotic" distance cut-off, as computed through Lloyd-Price et al. 2019 method.

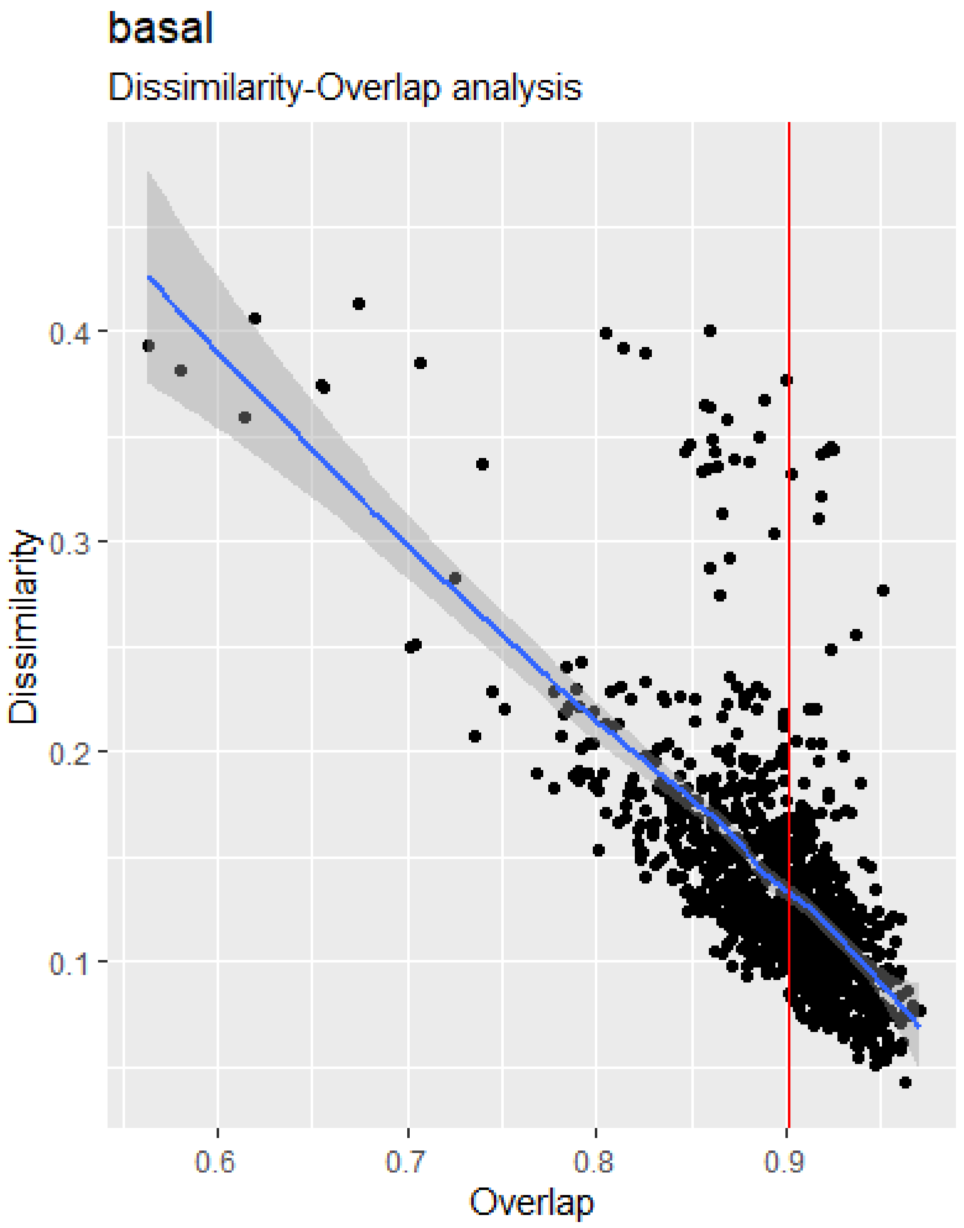
basal
Dissimilarity-Overlap analysis
Dissimilarity
0.4
0.3
0.2
0.1
0.6
0.7
0.8
0.9
Overlap

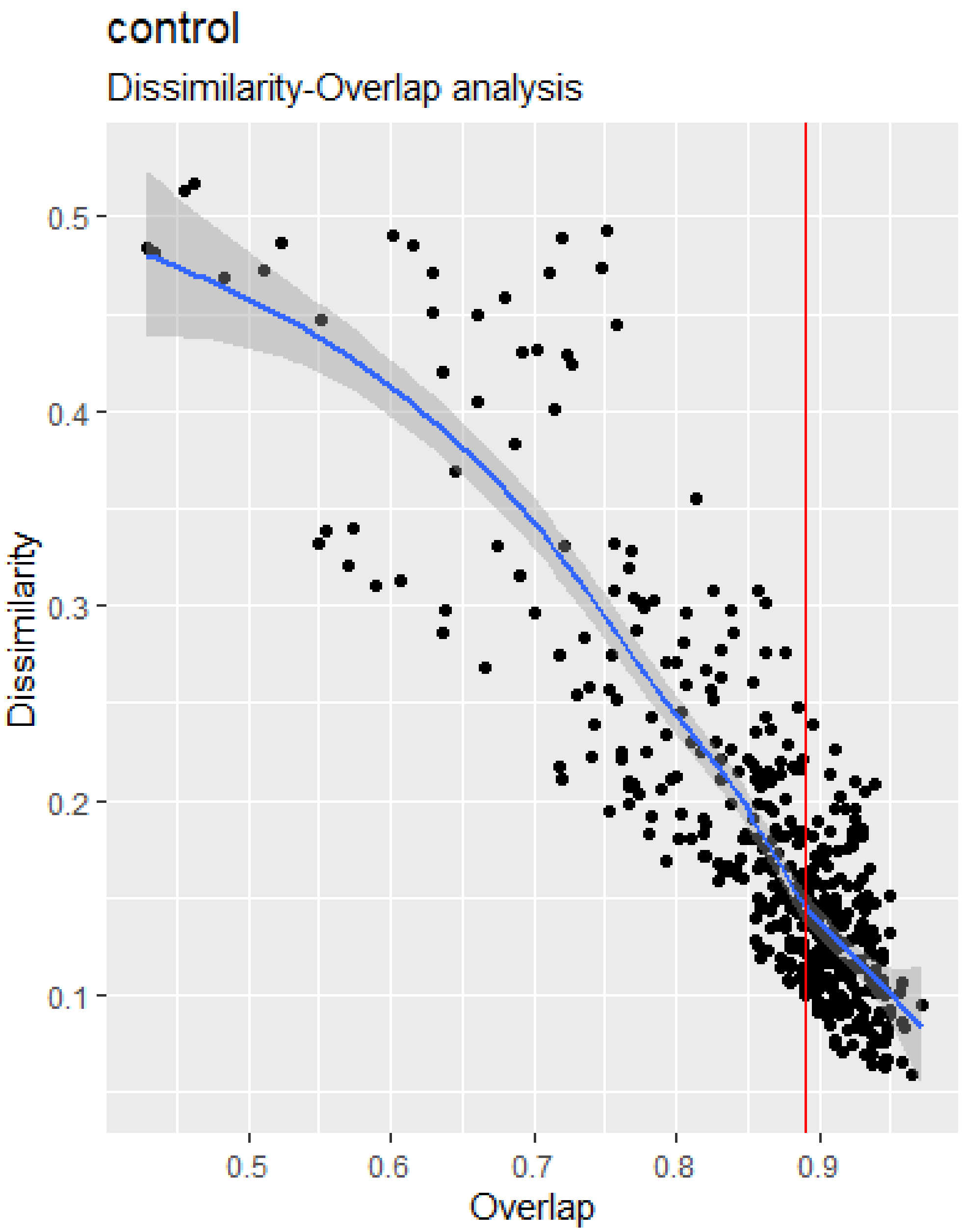

Figures S11. Scatterplots for the Dissimilarity vs. Overlap estimations within the CD ('Basal') and HC ('Control') sample sets. Analyses and plots were generated as described in https://microbiome.github.io/tutorials/DOC.html, by using the following R packages: Microbiome v.1.8.0, ggplot2_3.3.3 and Phyloseq_1.30.0. Smoothing method for regression curve: loess; vertical red lines represent the median Overlap value among sample pairs. Spearman correlation coefficient for pairwise Dissimilarity and Overlap distribution was -0.68 and -0.79 for the CD and HC sample sets, respectively. Negative slopes are indicative of a "healthy" microbial dynamics (Bashan et al. 2016).

Table S6. Results for the CLOUD test. Data shown were computed by using the Bray-Curtis dissimilarity matrix from QIIME2 diversity analysis, using dissimilarities between HC samples as "reference set" and setting "k" parameter = 5 (k nearest neighbours among the reference set with respect to the test sample). "K=5" represents approximately the 15% of reference sample size (total size = 31). "R_stat" indicate the sample-specific ratio between the test sample neighbourhood diameter and the average neighbourhood diameter in the reference set. P-values < 0.05 indicate samples that are significant "outlier" (bold characters).(Montassier et al. 2018).

SampleID	r_stat	p-value
**GF001**	0.5224	1
**GF006**	0.5521	1
**GF007**	0.9231	0.6444
**GF008**	0.7515	1
**GF009**	0.6429	1
**GF014**	**1.5993**	**0**
**GF015**	0.768	1
**GF023**	0.6015	1
**GF003**	0.7017	1
**GF005**	0.6771	1
**GF011**	0.7291	1
**GF013**	0.6272	1
**GF018**	0.7525	1
**GF019**	0.6516	1
**GF025**	0.6436	1
**GF028**	0.5545	1
**GF029**	0.7511	1
**GF031**	0.7444	1
**GF033**	0.7512	1
**GF017**	0.5282	1
**GF024**	0.5544	1
**GF026**	0.6714	1
**GF040**	1.0865	0.2
**GF041**	0.9355	0.6
**GF043**	0.7118	1
**GF054**	0.481	1
**GF060**	0.5927	1
**GF061**	0.6995	1
**GF063**	0.699	1
**GF100**	0.5171	1
**GF105**	0.7475	1
**GF106**	0.9049	0.7111
**GF108**	0.6412	1
**GF111**	0.5398	1
**GF002**	0.6393	1
**GF016**	0.5762	1
**GF020**	0.7138	1
**GF027**	0.7373	1
**GF032**	0.7715	1
**GF037**	0.7506	1
**GF044**	0.489	1
**GF048**	0.5494	1
**GF053**	0.632	1
**GF062**	0.9009	0.7556
**GF104**	0.751	1
**GF42**	0.7019	1
